# DREAMS-START (Dementia RElAted Manual for Sleep; STrAtegies for RelaTives) for people with dementia and sleep disturbances: a single-blind feasibility and acceptability randomized controlled trial

**DOI:** 10.1017/S1041610218000753

**Published:** 2018-09-17

**Authors:** Gill Livingston, Julie A. Barber, Kirsi M. Kinnunen, Lucy Webster, Simon D. Kyle, Claudia Cooper, Colin A. Espie, Brendan Hallam, Rossana Horsley, James Pickett, Penny Rapaport

**Affiliations:** 1 Division of Psychiatry, Faculty of Brain Sciences, UCL, London, UK; 2 Services for Ageing and Mental Health, Camden and Islington NHS Foundation Trust, London, UK; 3 Department of Statistical Science, Faculty of Mathematical & Physical Sciences, UCL, London, UK; 4 Sleep and Circadian Neuroscience Institute (SCNi), Nuffield Department of Clinical Neurosciences, University of Oxford, Oxford, UK; 5 Alzheimer's Society Research Network, London, UK; 6 Alzheimer's Society, London, UK

**Keywords:** sleep disorders, carers, cognitive behavioral therapy (CBT), randomized controlled trial (RCT), physical activity

## Abstract

**Background:**

40% of people with dementia have disturbed sleep but there are currently no known effective treatments. Studies of sleep hygiene and light therapy have not been powered to indicate feasibility and acceptability and have shown 40–50% retention. We tested the feasibility and acceptability of a six-session manualized evidence-based non-pharmacological therapy; Dementia RElAted Manual for Sleep; STrAtegies for RelaTives (DREAMS-START) for sleep disturbance in people with dementia.

**Methods:**

We conducted a parallel, two-armed, single-blind randomized trial and randomized 2:1 to intervention: Treatment as Usual. Eligible participants had dementia and sleep disturbances (scoring ≥4 on one Sleep Disorders Inventory item) and a family carer and were recruited from two London memory services and Join Dementia Research. Participants wore an actiwatch for two weeks pre-randomization. Trained, clinically supervised psychology graduates delivered DREAMS-START to carers randomized to intervention; covering Understanding sleep and dementia; Making a plan (incorporating actiwatch information, light exposure using a light box); Daytime activity and routine; Difficult night-time behaviors; Taking care of your own (carer's) sleep; and What works? Strategies for the future. Carers kept their manual, light box, and relaxation recordings post-intervention. Outcome assessment was masked to allocation. The co-primary outcomes were feasibility (≥50% eligible people consenting to the study) and acceptability (≥75% of intervention group attending ≥4 intervention sessions).

**Results:**

In total, 63out of 95 (66%; 95% CI: 56–76%) eligible referrals consented between 04/08/2016 and 24/03/2017; 62 (65%; 95% CI: 55–75%) were randomized, and 37 out of 42 (88%; 95% CI: 75–96%) adhered to the intervention.

**Conclusions:**

DREAM-START for sleep disorders in dementia is feasible and acceptable.

## Introduction

Currently, 47 million people live with dementia worldwide, with numbers expected to nearly triple by 2050 because of increasing longevity (Prince *et al.*, 2015). Sleep disturbances are common in dementia, for example, occurring in around 40% of people with Alzheimer's disease (AD) (Moran *et al.*, 2005; Dauvilliers, 2007; Zhao, 2016). Causes of sleep disturbance are varied and include pain relating to comorbid disorders, disorientation to time of day, or neuropsychiatric symptoms, including anxiety and depression. In addition, dementia may impair the sleep–wake cycle through degeneration of the suprachiasmatic nucleus, disrupting the normal circadian rhythm (Ju *et al.*, 2014). Sleep disruption reduces everyday function and quality of life (Kyle *et al.*, 2010). It also increases family carer burden, predicts depressive symptoms, and leads to care home admissions, thus increasing individual, societal, and economic costs associated with dementia (McCrae *et al.*, 2016; Livingston *et al.*, 2017).

There are, however, currently no known effective treatments for sleep problems in dementia, although there have been trials of drugs, including mirtazapine and melatonin which have been ineffective (McCleery *et al.*, 2014; Livingston *et al.*, 2017). Other studies incorporating sleep hygiene and light therapy have been too small for definitive results (McCurry *et al.*, 2009; Forbes *et al.*, 2014; Kinnunen *et al.*, 2017). Health teams use a mixture of sleep hygiene measures and psychotropic medication, extrapolated from other conditions, that give limited benefit, and medications may have side effects. Bright light therapy to strengthen circadian rhythmicity has some effect on sleep disturbances in the general population (McCurry and Ancoli-Israel, 2003). Cognitive behavioral techniques for sleep management have been effective in older adults without dementia and in family carers of people with dementia (Montgomery and Dennis, 2003; Sivertsen and Nordhus, 2007).

The objective of this study is to test the feasibility and acceptability of Dementia RElAted Manual for Sleep; STrAtegies for RelaTives (DREAMS-START), a multicomponent manualized intervention built on the above available evidence, to manage clinically significant sleep disturbance in people with dementia living in their own homes.

## Methods

### Study design and participants

This is a two-armed randomized controlled trial, recruiting from three UK sites; Camden and Islington NHS Foundation Trust, Barnet, Enfield and Haringey Mental Health NHS Trust, and Join Dementia Research (JDR), where people register their interest in participating in dementia research. London – Queen Square Research Ethics Committee (Reference: 16/LO/0670) approved the study. We obtained written consent from all participating family carers and patients with the mental capacity to give informed consent to participation in the trial prior to their enrollment. If the person with dementia did not have capacity to give consent, we required a consultee's declaration. The trial steering committee provided overall supervision of the trial with an independent chairperson leading it. The full protocol is in the Appendix (see online supplementary material).

Eligible patients from the trusts lived in their own home, had a clinical diagnosis of dementia and a sleep disorders inventory (SDI) item score ≥4 (Tractenberg *et al.*, 2003), judged as problematic by the person with dementia or their family, and had a primary family carer who provided support at least weekly. People with dementia, who had a primary sleep disorder diagnosis, such as sleep apnoea, were excluded. Patients and carers were assessed at home after consent and at follow-up three months later. The baseline measures included socio-demographic details and dementia severity through the Clinical Dementia Rating (CDR), a reliable and valid instrument for classifying clinically diagnosed dementia (Hughes *et al.*, 1982; Morris, 1997) as very mild (0.5), mild (1), moderate (2) or severe (3). After enrollment, we asked patients to wear wrist-worn actiwatches (MotionWatch 8) CamNtech Ltd. 24 h a day for two weeks before randomization and again at follow-up.

### Randomization and masking

An independent statistician produced computer-generated randomization lists stratified by site and based on random permuted blocks of sizes three and six to allow 2:1 allocation to intervention: Treatment as Usual (TAU) groups. Therapists worked in two separate teams of two therapists each and assessed outcomes for participants, to whom the other team had delivered the intervention, masked to group allocation. A third non-therapist researcher assessing outcomes was masked to all allocations. Due to the intervention's nature it was not possible to mask the trial participants. When arranging follow-up, participants were reminded not to disclose their allocation group to the assessor, and to remove from view anything related to DREAMS-START.

### Procedures

**Figure 1.**
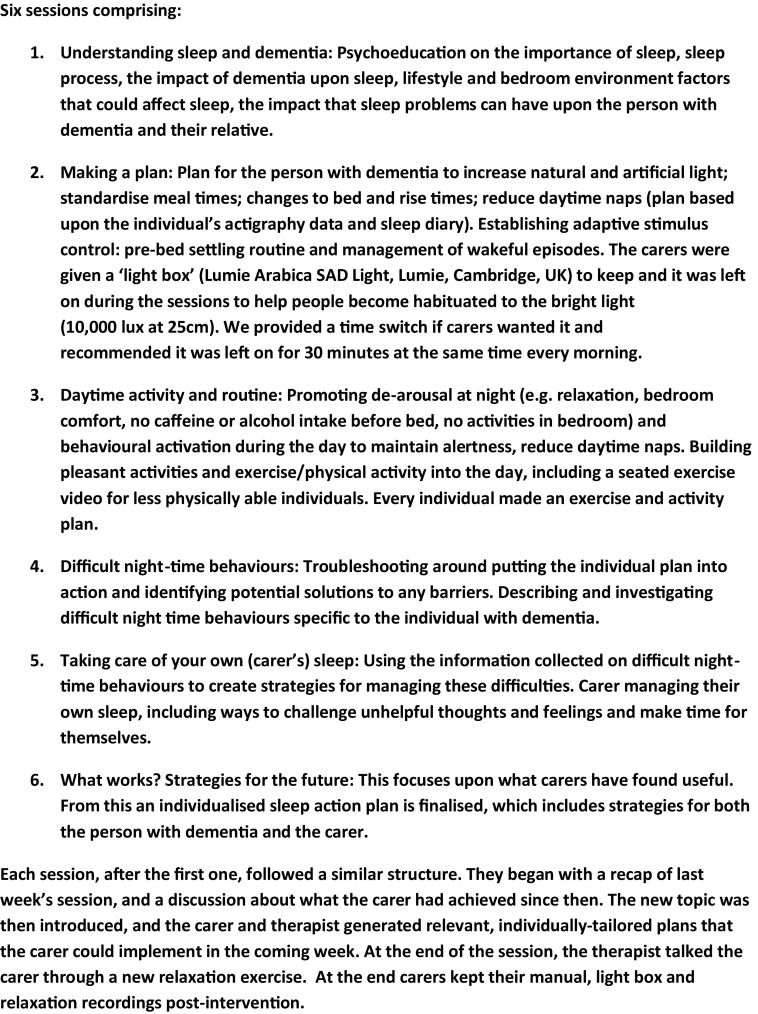
Structure of DREAMS-START manual.

Those allocated to the intervention received the six-session DREAMS-START manual. We developed DREAMS-START for the study in an iterative co-production process, involving dementia and sleep experts and Public and Patient Input (PPI) (PR, GL, SK, CE, CC with PPI led by RH and JP). We incorporated prior evidence, some existing materials and used the START manual structure (Livingston *et al.*, 2013; Livingston *et al.*, 2014). This iterative coproduction is reported in detail elsewhere (Kinnunen *et al.*, 2017). DREAMS-START comprised cognitive-behavioral components, including psychoeducation, light therapy, establishing a new sleep–wake schedule (based on review of actiwatch data), behavioral activation, relaxation, and coping skills for families (see, Figure 1 for details of DREAMS-START). Trained and clinically supervised psychology graduates delivered it to carers at home, unless the carer requested to have sessions elsewhere. Each session lasted about 1 h, and took place approximately weekly, at a time convenient to the carer. The sessions were delivered to the family carer, unless a paid carer was with the person around the clock, and therefore would be most likely to implement strategies. We encouraged the carers to have the sessions by themselves, so they could talk without potentially distressing the person with dementia. However, if people with dementia wanted to participate they were included in the sessions.

Our clinical psychologist, PR, met each team of therapists for 1.5 h of group supervision fortnightly. Additionally, she was available for individual supervision, requested by the psychology graduates on an *ad hoc* basis, or occasionally initiated by the investigators. If the therapists had any urgent concerns they approached PR, CC, or GL (e.g. if risk issues arose). The group supervision format was to ensure good case management, clinical skills development and safe practice, and to maximize peer support.

PR devised a fidelity checklist comprising the most important components of each session. Therapist's audio-recorded one session selected at random by the trial manager per participant. The other therapist in the same team then rated the recording for “keeping the session to time,” “keeping the carer focused on the manual,” “keeping the carer engaged in the session,” and “managing the concerns of the carer.” Scores ranged from 1 “not at all” to 5 “very focused.”

All participants received TAU. This was expected to vary between trusts and patients, but be in line with the NICE dementia guidelines (NICE/SCIE, 2006), consisting of assessment, diagnosis, symptomatic interventions, risk assessment and management, and information. These included medication; cognitive stimulation therapy; neuropsychiatric symptoms treatment; driving advice; medical identification (ID) bracelets; capacity assessment and advice regarding power of attorney; referral to dementia advisors/navigators for signposting and to social services for personal care, tableware, day center and financial advice, and carer support (START in some trusts). We also gathered details of services the JDR volunteers received.

After the follow-up data had been collected, the control group participants received a summary of the baseline actiwatch data, with advice on improving sleep.

### Outcomes

Trained research team member, masked to group assignment, assessed patients for outcomes. The primary outcomes were feasibility of recruitment and treatment adherence. These were assessed by (1) the proportion of eligible carers consented; (2) the proportion randomized after baseline assessment; and (3) acceptability of DREAMS-START by adherence. The secondary outcomes were referral rates; follow-up rates at three months; all psychotropic medication prescription (to define rescue medication's role); reported side effects: co-morbid physical illnesses and patient falls; and feasibility and acceptability of interview schedules and actigraphy assessed through instrument completion rates.

We collected sleep measures (interview and actigraphy) before randomization and three months after. Actigraphy estimates sleep and wakefulness from movement and time, and has been used previously in a small trial to improve sleep for people with dementia (McCurry *et al.*, 2005). We asked carers to define each participant's sleep analysis window, either by recording the person with dementia's bed and rise times in a sleep diary, or by pressing the actiwatch's “event marker” button. The SDI (Tractenberg *et al.*, 2003) is a valid and reliable seven-item measure of a person with dementia's sleep disturbance. Scores ≥4 on individual items equate to clinically significant sleep disturbance and study eligibility. The SDI total (sum of items) score ranges from 0 to 84. The alternative SDI mean global score is calculated as the mean frequency × mean severity (range 0–12). Daytime sleepiness was measured using the validated eight-item Epworth Sleepiness Scale (ESS) (Johns, 1994; Spira *et al.*, 2012). Possible scores range 0–24, with >10 indicating excessive sleepiness. We measured overall neuropsychiatric symptoms using the neuropsychiatric inventory (NPI) (Cummings *et al.*, 1994) at baseline and three months. This validated instrument has 12 neuropsychiatric symptoms domains (scored 0–12). The possible highest score is 144. Higher scores mean increasing severity. Quality of life was measured using the DEMQOL-Proxy (Smith *et al.*, 2007), a 31-item interviewer-administered valid and reliable instrument for dementia, which can be used with the Client Service Receipt Inventory to calculate cost-effectiveness (Mulhern *et al.*, 2012).

We collected data on the family carers, including sleep, commonly disrupted by person with dementia's sleep–wake patterns, using the validated, reliable Pittsburgh Sleep Quality Index (PSQI) (Buysse *et al.*, 1989), and the Sleep Condition Indicator (SCI) (Espie *et al.*, 2014), an eight-item scale, characterizing sleep both dimensionally and against insomnia disorder criteria. Mood was measured using the validated, reliable Hospital Anxiety and Depression Scale (HADS) (Bjelland *et al.*, 2002; Snaith, 2003), and subjective burden using the validated Zarit Burden Interview (ZBI) (Zarit *et al.*, 1980). Carer's quality of life was measured on the Health Status Questionnaire-12 (HSQ-12), a 12-item scale (Pettit *et al.*, 2001; Barry *et al.*, 2007).

### Sample size

We estimated that with 40 intervention participants (larger to allow a more precise estimate of proportion adhering to intervention) and 20 controls, we would achieve the following 95% confidence interval (CI) for our expected adherence and participation estimates:

1. Proportion of participants adhering to intervention – expected value 75%, 95% CI: 59–87%
2. Proportion of appropriate referrals consenting to the trial – expected value 50%, 95% CI: 41–59%

This sample size was also judged as sufficient for estimating the standard deviation as required for the sample size calculation in the main trial. We envisaged in our protocol that it would be actigraphy but that we would be open to change this from information in the trial, and we would also have information to calculate sample size using the scales in the trial. Our estimated recruitment referral rate was approximately six potential participants per week. Two out of these were expected to be suitable and agree to participate. Our expected follow-up rate was approximately 80%.

The 95% CI for expected adherence and participation estimates would provide acceptable ranges to inform continuation to the main trial. We specified our “stop–go” measures would be related to the proportion adhering, with ≥70% meaning proceed to a main trial.

### Analysis

For all eligible patients and carers, we summarized recruitment site and sex of the person with dementia, and carer sex and relationship to the person with dementia comparing those who consented with those who did not. We calculated with 95% CI: proportion of screened patients who were eligible for the trial, proportion of eligible referrals consenting to the trial, proportion of participants in each randomized group who dropped out or were lost to follow-up, proportion of participants in the intervention group who adhered to DREAMS-START (attended a ≥4/6 sessions), median number of sessions attended by those in the intervention group. The frequency (%) of participants in each randomized group who had taken psychotropic drugs and melatonin during the three months prior to the baseline and follow-up was calculated. Frequency (%) of co-morbid physical illnesses and patient falls was summarized by randomized group at baseline and three months.

We summarized socio-demographic characteristics and baseline and follow-up actigraphy measures and other scores using means (with standard deviations), medians (with interquartile ranges, IQR), counts and proportions, as appropriate. The actigraphy data were analyzed using MotionWare Software 1.1.25. We defined each sleep period, using the bed and rise times in the sleep diary or by event markers. We used the carer's verbal report when neither was available. When unsure, two researchers edited a sleep period and reached a consensus. We removed from non-parametric circadian rhythm analysis (NPCRA) periods of missing data >3 h and excluded sleep data based on fewer than seven nights. For exploratory sleep analyses, we defined “core night-time” as midnight to 6 am.

The number of participants with available data was summarized for each outcome.

Follow-up carer and patient questionnaire scores were compared between randomized groups using regression models to provide estimates of the effect of DREAMS-START with 95% CI (e.g. difference in means), adjusted for baseline score and site. Ordinary least squares regression was used except for SDI scores where assumptions of the model were violated and quantile (median) regression methods were used. Formal analysis for sleep measures at 3 months was focused on sleep efficiency (time asleep\time in bed), relative amplitude, activity count for most restful hours and activity count for most active hours (predefined in analysis plan). Regression models were adjusted for baseline score and site. Ordinary least squares were used where appropriate, otherwise estimates were obtained from quantile (median) regression.

## Results

### Participant recruitment and flow

**Figure 2.**
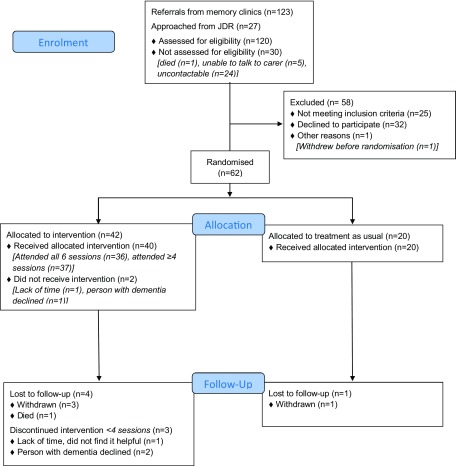
CONSORT flow diagram: Summary of recruitment and follow-up.

The flow of participants through the trial is described using a CONSORT diagram (Figure 2). We were referred 123 people through memory clinics from 04/08/2016 to 03/04/2017. We stopped recruiting when we were confident that we would have sufficient randomized participants. The study was open to JDR recruitment from 22/11/2016 to 24/03/2017. We found 140 potential people in JDR, who lived in the area and had registered as having dementia and a family supporter, and contacted 27 using their preferred means (25 by email; 2 by phone) from 05/12/2016 to 21/03/2017. We followed up email by phone calls; 19 did not respond, 3 were ineligible (2 had no sleep problems; 1 was moving out of London), for one person we could only speak to the person with dementia and were unable to determine eligibility. 95/120 (79%; 95% CI 71–86%) people assessed were eligible.

### Comparison of those who consented with those who did not

Table 1 compares consenters and non-consenters demographic details and shows the study had good external validity. The care recipients who did not consent were, however, more often male.

**Table 1.** Characteristics of those eligible but did not consent vs. those who consented and were randomized

	person with	not consented	randomized	p-value
	dementia	(*n* = 33)	(*n* = 62)	(*χ*^2^ test)
Site	Camden and Islington	24 (73%)	40 (65%)	
	Barnet, Enfield &	7 (21%)	20 (32%)	
	Haringey			
	Join Dementia Research	2 (6%)	2 (3%)	p = 0.46
Sex	Male	20 (61%)	19 (31%)	
	Female	13 (39%)	43 (69%)	p = 0.008
carer		not consented	randomized	
		(*n* = 33)	(*n* = 62)	
Carer sex	Male	11 (33%)	18 (29%)	
	Female	22 (67%)	44 (71%)	p = 0.67
Relationship to person	Spouse	9 (28%)	19 (31%)	
with dementia				
	Child/child in law	22 (69%)	41 (66%)	(spouse vs. other)
	Other	1 (3%)	2 (3%)	p = 0.80

Table 2 compares demographic and diagnostic details of the randomized groups. Overall there was a good demographic mix with recruitment from a range of ethnicities, age groups and relationship between the person with dementia and their carer. There was a range of diagnoses with most people having AD or a mixed dementia. Most primary carers (45; 73%) lived with the person with dementia, 4 lived with another family member, so 49/62 (79%) lived with family members, 6 (10%) had paid carers living with them, and 7 (11%) had family carers but lived alone. The carers in the intervention group included more women, were younger and less likely to be co-residents or spouses. The people with dementia in the intervention group were more likely to be women and more had a diagnosis of AD than in the TAU group.

**Table 2.** Baseline demographic data and diagnostic characteristics by randomized group

person with dementia		intervention (*n* = 42)	TAU (*n* = 20)
Site	Camden and Islington	27 (64%)	13 (65%)
	Barnet, Enfield & Haringey	14 (33%)	6 (30%)
	Join Dementia Research	1 (2%)	1 (5%)
Sex	Male	9 (21%)	10 (50%)
	Female	33 (79%)	10 (50%)
Age (years)	Mean (SD)	80.4 (9.0)	79.6 (7.0)
Lived with/alone	Family carer	28 (67%)	17 (85%)
	Another family member	3 (7%)	1 (5%)
	Paid carer	5 (12%)	1 (5%)
	Alone	6 (14%)	1 (5%)
Diagnosis	Alzheimer's disease	22 (52%)	7 (35%)
	Dementia with Lewy bodies	2 (5%)	3 (15%)
	Mixed	8 (19%)	4 (20%)
	Vascular dementia	8 (19%)	6 (30%)
	Alcohol-related dementia	1 (2%)	0 (0%)
	Unspecified	1 (2%)	0 (0%)
Age left education (years)	Mean (SD)	15.7 (3.7) (*n* = 40)	16.4 (4.9) (*n* = 19)
Marital status	Single	1 (2%)	1 (5%)
	Married	17 (40%)	11 (55%)
	Divorced	1 (2%)	0 (0%)
	Widowed	23 (55%)	8 (40%)
Ethnic group	White	27 (64%)	13 (65%)
	Asian	3 (7%)	3 (15%)
	Black	7 (17%)	2 (10%)
	Other	5 (12%)	2 (10%)
CDR global	Very mild	5 (12%)	3 (15%)
	Mild	16 (38%)	6 (30%)
	Moderate	18 (43%)	9 (45%)
	Severe	3 (7%)	2 (10%)
carer		intervention (*n* = 42)	tau (*n* = 20)
Carer sex	Male	10 (24%)	8 (40%)
	Female	32 (76%)	12 (60%)
Carer age (years)	Mean (SD)	56.15 (13.54)	59.09 (12.22)
Family carer co-resident	Yes	28 (67%)	17 (85%)
Relationship to person with dementia	Spouse	10 (24%)	9 (45%)
	Child/child in law	30 (72%)	11 (55%)
	Grandchild	1 (2%)	0
	Friend	1 (2%)	0
Carer ethnicity	White	29 (69%)	14 (70%)
	Asian	3 (7%)	3 (15%)
	Black	7 (17%)	1 (5%)
	Other	3 (7%)	2 (10%)

*Notes:* The numbers are frequency (%) unless otherwise stated.

### Primary outcomes

63 (66%; 95% CI 56–76%) of eligible referrals consented (61 from memory clinics and 2 from JDR) and 62/95 (65%; 95% CI 55–75%) of whom were randomized. In 8 cases, we consented two carers who were involved in the intervention (3 people had two family carers; 5 had one family, and 1 paid carer).

### Intervention adherence and delivery

We randomized 42 people to DREAMS-START, 2 dropped out before commencement. The median number of sessions attended was 6; 37 (88%, 95% CI 75–96%) attended ≥4 and 3 (7%) attended 1–3 sessions. Intervention delivery duration was a median of 49.5 days (IQR = 43–64.5). On average, each session took 69 min. The intervention was delivered by four trained and supervised psychology graduates, two women and two men, who were ethnically white British (3) and Asian British (1). Their ages ranged from 23–33 years. The therapists visited all but two participants in their homes; one was seen for one session at UCL and another for all sessions at a Memory Service.

Sessions were delivered to 21 carers without the person with dementia present; comprising 18 interventions with the family carer alone, 2 interventions with paid carers (1 with one paid carer and 1 with three paid carers), and 1 delivered to a family carer and a paid carer together. In 5/21 of these interventions, the person with dementia was present in the same room, but did not participate as they did not want to or could not (due to auditory or cognitive impairment).

For 13 dyads, both the person with dementia and their carer attended every session. In others, the person with dementia participated in some sessions: 12 interventions were delivered with the person with dementia and their family carer and 1 intervention with the person with dementia, their family carer and paid carer. One person with dementia participated in all sessions, but their family carer only in three.

Fidelity was rated for 34/40 (85%) participants who started DREAMS-START; three refused recording, two stopped DREAMS-START before the planned recording, and one recording was partial and unscorable. Managing the carer's concerns and keeping the carer engaged in the session median score was 5/5 (IQRs: 4–5 and 4.25–5, respectively), keeping the carer focused on the manual and keeping to time was rated 4/5 (IQRs: 4–5 and 3.25–5).

## Secondary outcomes

### Referral rates

Four potential participants were referred by the memory clinics weekly.

### Follow-up rates

57/62 (92%) of those randomized were followed up at 3 months. Loss to follow-up in the intervention group was 4/42 (9.5%; exact 95% CI 3–23%) and in the TAU group 1/20 (5%; exact 95% CI 0.1–25%): two withdrew consent, two were uncontactable, and one person with dementia died (Figure 2).

### All psychotropic medication prescription

At baseline and 3 month follow-up, 19 (45%) and 16 (43%) of the intervention group received at least 1 prescription of psychotropic medication or melatonin, respectively. The corresponding TAU numbers were 9 (45%) and 8 (44%).

### Comorbid illness and side-effects

Table 3 shows comorbid illnesses at baseline and comorbid illness and possible side effects at three months. There is no clear difference between the groups.

**Table 3.** Summary of comorbid illness and side effects by randomized group

		intervention	tau
Baseline		(*N* = 42)	(*N* = 20)
	Falls	17 (40%)	7 (35%)
	Gastro	21 (50%)	9 (45%)
	Neurological	19 (45%)	10 (50%)
	Infections	14 (34%)	6 (30%)
	Other	6 (14%)	2 (11%)
3 Months		(*N* = 38)	(*N* = 18)
	Falls	16 (42%)	7 (39%)
	Gastro	16 (42%)	4 (22%)
	Neurological	15 (39%)	10 (56%)
	Infections	18 (47%)	5 (28%)
	Other	9 (24%)	9 (50%)

*Notes:* The numbers are frequency (%).

### Potential outcomes for the main trial

Table 4 summarizes scores of patient and carer questionnaires at baseline and 3 months and indicates numbers with available data for each. At baseline, the NPI, PSQI, SCI, and HSQ were completed for 61/62 (98.4%). Other measures were 100% completed. Summary scores are similar between the two groups, although carers in the intervention group had consistently better scores than TAU.

**Table 4.** Completion rates and scores of baseline and three month follow-up patient and carer validated interview measures by randomized group

person with dementia baseline			intervention (*n* = 42)	tau (*n* = 20)	
SDI global score		Median (IQR)	2.57 (1.35 to 3.67)	2.98 (1.85 to 3.96)	
SDI total score		Median (IQR)	26.5 (19 to 36)	32.5 (20.5 to 40)	
ESS total score		Mean (SD)	9.95 (6.02)	8.85 (6.31)	
NPI total score		Mean (SD)	42.02 (23.17)	46.90 (23.48)	
			(*N* = 41)		
DEMQOL-Proxy		Mean (SD)	87.57 (10.73)	88.51 (10.14)	
carer baseline		intervention (*n* = 42)	tau (*n* = 20)		
PSQI global score		Mean (SD)	9.22 (4.08) (*N* = 41)	10.40 (4.52)	
SCI total score		Mean (SD)	15.32 (8.22) (*N* = 41)	13.50 (5.92)	
HADS scores		Anxiety: Mean (SD)	8.17 (4.66)	9.30 (3.80)	
		Depression: Mean (SD)	5.24 (4.33)	7.65 (4.60)	
		Total: Mean (SD)	13.40 (8.35)	17.05 (7.80)	
ZBI score		Mean (SD)	37.69 (18.39)	38.30 (19.27)	
HSQ scores		Physical health: Mean (SD)	67.89 (32.79) (*N* = 41)	52.50 (39.47)	
		Mental health: Mean (SD)	60.95 (23.17)	52.33 (24.02)	
person with dementia three month			intervention (*n* = 42)	tau (*n* = 20)	adjusted tr effect (i-tau) (95% ci)^b^
SDI global score		Median (IQR)	0.92 (0.49 to 2.94)	2.43 (0.82 to 3.88)	−0.30 (−1.42 to 0.82)^a^
SDI total score 0 to 84		Median (IQR)	16 (9 to 29) (*N* = 38)	30 (14 to 37) (*N* = 18)	−7 (−17.53 to 3.53)^a^
ESS total score		Mean (SD)	7.17 (5.87) (*N* = 36)	9.00 (7.55) (*N* = 18)	−2.86 (−5.54 to −0.17) (*N* = 54)
DEMQOL-Proxy		Mean (SD)	93.52 (10.12) (*N* = 37)	87.07 (10.22) (*N* = 18)	7.08 (2.25 to 11.91) (*N* = 55)
NPI total score		Mean (SD)	38.69 (23.57) (*N* = 36)	44.72 (23.22) (*N* = 18)	−1.99 (−11.66 to 7.68) (*N* = 54)
carer three months			intervention (*n* = 42)	tau (*n* = 20)	adjusted tr effect (i-tau) (95% ci)^b^
PSQI global score		Mean (SD)	9.37 (4.16) (*N* = 38)	9.5 (4.49) (*N* = 18)	1.03 (−1.05 to 3.11) (*N* = 55)
SCI total score		Mean (SD)	15.45 (7.45) (*N* = 38)	14.53 (8.54) (*N* = 19)	−0.41 (−3.75 to 2.93) (*N* = 56)
HADS scores	Anxiety	Mean (SD)	8.76 (5.57)	9.05 (4.22)	1.13 (−0.31 to 2.56)
	Depression	Mean (SD)	5.71 (4.43)	8.79 (4.88)	−1.05 (−3.01 to 0.91)
	Total	Mean (SD)	14.47 (9.20) (*N* = 38)	17.84 (8.43) (*N* = 19)	0.51 (−2.39 to 3.42) (*N* = 57)
ZBI score		Mean (SD)	36.5 (17.07) (*N* = 38)	42.16 (16.45) (*N* = 19)	−5.32 (−9.83 to −0.82) (*N* = 57)
HSQ scores	Physical health	Mean (SD)	68.42 (32.60) (*N* = 38)	54.39 (35.94) (*N* = 19)	3.12 (−12.27 to 18.52) (*N* = 57)
	Mental health	Mean (SD)	55.96 (26.19) (*N* = 38)	48.07 (21.15) (*N* = 19)	1.25 (−9.52 to 12.02) (*N* = 57)

IQR = interquartile range; SD = standard deviation.

^a^Quantile (median) regression.

^b^Estimates are from models adjusted for baseline score and site. Regression is OLS unless otherwise indicated.

Over 90% of those randomized completed the SDI, HADS, ZBI, SCI, and the HSQ at 3 months. Over 85% completed the other questionnaires. Summary data indicate generally better scores for the intervention group for both patient and carer measures. After adjusting for site and baseline score, statistically significant improvements due to intervention are evident for ESS, DEMQOL-Proxy, and ZBI.

Table 5 shows baseline sleep data for those wearing the actiwatches for ≥7 nights with complete data (i.e. not including 24-h periods with >3 h of missing data). Only 1/62 participants with dementia randomized did not wear the watch for ≥7 nights at baseline. The sleep diary or event markers were used by 50/62 (81%) of randomized participants to record the person with dementia's bed and rise times. Of the remaining 12, 8 (13%) gave a verbal report of sleep pattern, while 4 (6%) did not. Table 6 shows the follow-up sleep data. Overall, there was follow-up data for 49/62 (79%) randomized; 6/57 (10.5%) randomized and still in the study refused to wear the watch again; 49/51 (96%) of those who did wear it had actigraphy data for at least seven days. Carers of 42 (82%) of these participants provided their relative's bed and rise times via the sleep diary or event markers. Of the remaining 11, eight (16%) provided a verbal report of their relative's sleep pattern.

**Table 5.** Baseline sleep and non-parametric circadian rhythm analysis measures by randomized group

		intervention	tau
sleep measures		*n* = 41	*n* = 20
Sleep efficiency (%)^a^	Median (IQR)	76.90 (66.60 to 83.00)	80.68 (70.75 to 86.30)
Average sleep time (min)	Median (IQR)	448.00 (376 to 503)	442.00 (388 to 568.50)
Average wake time (min)	Median (IQR)	111 (77 to 152)	114 (58.5 to 147.5)
Average time of lights out/bedtime (24 h)^b^	Median (IQR)	22:26 (22:07 to 23:08)	22:25 (21:37 to 23:25)
Average time of falling asleep (24 h)^b^	Median (IQR)	22:43 (22:07 to 23:40)	22:45 (22:04 to 00:16)
Average time of waking up (24 h)	Median (IQR)	08:10 (07:41 to 8:54)	08:20 (07:48 to 8:46)
Average time of getting up (24 h)	Median (IQR)	08:14 (07:52 to 09:00)	08:23 (07:56 to 08:56)
Average time in bed (h)	Mean (SD)	9.91 (1.35)	10.01 (1.80)
non-parametric circadian rhythm analysis measures		*n* = 42	*n* = 20
Relative amplitude	Median (IQR)	0.71 (0.48 to 0.82)	0.78 (0.56 to 0.90)
Inter-daily stability	Mean (SD)	0.38 (0.16)	0.43 (0.17)
Intra-daily variability	Mean (SD)	1.11 (0.40)	1.03 (0.32)
L5 – average activity count for five most restful hours	Median (IQR)	1381 (572 to 1919)	761 (358.5 to 1616)
Start hour of five most restful hours (24 h)^b^	Median (IQR)	01:00 (00:00 to 03:00)	01:00 (00:00 to 02:00)
	Median (IQR)	8155 (5127 to 12281)	8212.0 (3730.5 to 14545.5)
Start hour of ten most active hours (24 h)	Median (IQR)	10:00 (8:00 to 12:00)	8:30 (8:00 to 11:00)
core night-time sleep measures		*n* = 41	*n* = 20
Sleep efficiency (%)^a^	Median (IQR)	76.4 (68.1 to 83.9)	81.3 (67.15 to 91.05)
Average sleep time (min)	Median (IQR)	278 (245 to 303) (*N* = 39)	300 (246 to 331) (*N* = 19)
Average wake time (min)	Median (IQR)	57 (47 to 75) (*N* = 39)	52 (26 to 76) (*N* = 19)

*Notes:* Sleep and core night-time measures are for those ≥7 nights of data available. Non-parametric circadian rhythm analysis (NPCRA) data are those calculated with all 24-h periods with >3 h of missing data excluded.

IQR = interquartile range; SD = standard deviation.

^a^Time asleep\time in bed.

^b^To calculate summaries, times have been ordered from midday one day until midday the next day.

**Table 6.** Three-month sleep and non-parametric circadian rhythm analysis measures by randomized group

sleep measures	*n* = 32	intervention *n* = 17	tau *n* = 49	adjusted tr effect (i-tau) (95% ci)^a^
Sleep efficiency (%)^b^	Median (IQR)	76.05 (65.0 to 81.9)	78.6 (72.1 to 82.3)	0.39 (−4.89 to 5.68)^c^
Average sleep time (min)	Median (IQR)	418.5 (383.5 to 506)	475 (396 to 534)	
Average wake time (min)	Median (IQR)	112.5 (94.5 to 157.5)	105 (88 to 133)	
Average time of lights out/bedtime (24h)^d^	Median (IQR)	22:15 (21:36 to 22:50)	22:03 (21:21 to 23:28)	
Average time of falling asleep (24 h)^d^	Median (IQR)	22:49 (21:59 to 23:23)	22:22 (21:39 to 23:55)	
Average time of waking up (24 h)	Median (IQR)	08:04 (07:16 to 08:38)	08:09 (07:38 to 08:41)	
Average time of getting up (24 h)	Median (IQR)	08:06 (07:20 to 08:44)	08:17 (07:48 to 08:43)	
Average time in bed (h)	Mean (SD)	9.89 (1.83)	10.05 (1.83)	
non-parametric circadian rhythm analysis measures		*n* = 34	*n* = 17	*n* = 51
Relative amplitude	Median (IQR)	0.71 (0.49 to 0.87)	0.76 (0.51 to 0.90)	−0.02 (−0.10 to 0.06)^c^
Inter-daily stability	Mean (SD)	0.38 (0.18)	0.47 (0.19)	
Intra-daily variability	Mean (SD)	1.04 (0.38)	0.98 (0.40)	
L5 – average activity count for five most restful hours	Median (IQR)	1067 (621 to 2003)	981 (493 to 1940)	76.88 (−521.40 to 675.16)^c^
Start hour of five most restful hours (24h)^d^	Median (IQR)	01:00 (00:00 to 03:00)	01:00 (01:00 to 02:00)	
M10 – average activity count for 10 most active hours	Median (IQR)	8247 (4258 to 12155)	8132 (5960 to 17158)	−198.29 (−1717.83 to 1321.25)^c^
Start hour of ten most active hours (24 h)	Median (IQR)	10:00 (8:00 to 11:00)	9:00 (8:00 to 10:00)	
core night-time sleep measures		*n* = 32	*n* = 17	
Sleep efficiency (%)^b^	Median (IQR)	79.0 (63.75 to 82.6)	80.9 (69.8 to 86.5)	
Average sleep time (min)	Median (IQR)	285 (243 to 303) (*N* = 30)	291 (251 to 311) (*N* = 17)	
Average wake time (min)	Median (IQR)	62 (43 to 80) (*N* = 30)	49 (39 to 70) (*N* = 17)	

*Notes:* Sleep and core night-time measures are only for those with at least seven nights of data available. NPCRA (non-parametric circadian rhythm analysis) data are those calculated with all 24-h periods with >3 h of missing data excluded.

IQR = interquartile range; SD = standard deviation.

^a^Estimates are from models adjusted for baseline score and site. Regression is OLS unless otherwise indicated.

^b^Time asleep\time in bed.

^c^Quantile (median) regression.

^d^To calculate summaries, times have been ordered as night time i.e. from midday to midday the following day.

## Discussion

This is the first non-pharmacological randomized controlled trial for sleep problems in dementia powered to find if the intervention was feasible and acceptable. It met predetermined criteria to proceed to a full trial. It was not powered for efficacy and our efficacy results are indicative rather than definitive. One measure of acceptability is the adherence rates to the intervention, and a good adherence rate is a matter of judgment, with clinical trials of medication for people being treated for chronic illnesses reporting rates of 43–78% (Osterberg and Blaschke, 2005). Our predetermined level in this trial was a relatively high rate of 75%, which was exceeded. In addition, most of those in the intervention group found it acceptable in that they attended all sessions once they had started.

This trial shows that it is possible to deliver a complex manual-based intervention and measures fidelity. These results indicate that it should be possible to deliver the intervention reliably in future. High fidelity was achieved measured through predetermined fidelity check lists and sessions were recorded and fidelity measured. While each possible intervention was mentioned, therapists and carers were explicitly told that if something was not applicable then to mention it as a principle but not go into detail or make it a goal e.g. if someone was wheelchair bound not to suggest they went out walking daily. Recruitment rates and consent were high from those referred by health professionals but we found that a research register may be a less fruitful source of recruitment possibly because people may register and then their circumstances may change. Generally, 80% follow-up or above is regarded as satisfactory, and we achieved 92%. JDR did not meet our criteria for recruitment.

At follow-up, less than 80% of people randomized had enough actigraphy data to analyze and only 68% had sleep diary/event marker data. We originally envisaged that actigraphy data would be the full trial primary outcome, but we are unlikely to have the level of data required for this. Families thought the person they looked after was sleeping better, but the actigraphy results did not support this. Actigraphy infers “sleep” from lack of activity during the sleep window. Movement is in contrast directly measured. There is little actigraphy validation data in people with dementia, who may frequently stay still while awake or may sleep during the day. It has been validated against polysomnography (PSG), with good accuracy at detecting sleep (96.5%), but not at detecting wakefulness (32.9% accuracy) throughout the age range. Validity worsened with increasing age (Marino *et al.*, 2013). In older women without dementia (mean age 69 years) actigraphy results were unacceptable for those with low sleep efficiency (Taibi *et al.*, 2013). The algorithms are also not designed to detect daytime sleepiness. This suggests that it is important that better methods of measuring sleep in people with dementia are measured and it is possible that WiFi enabled headbands may work better. There was, however, indications from actigraphy, that intervention participants were more active during the day and less active at night. These accord with the validated instruments results.

We achieved high completion rates of the questionnaire measures using carers as informants. The scores on the instruments are useful to inform the design of a full trial. The SDI was completed at follow-up by 90% of those initially recruited to the trial. This appeared to be the most practical way to measure sleep for future studies in this area. Summary data for the carer-reported instruments indicated generally better scores for the intervention group, including a significant improvement in both quality of life of people with dementia and daytime sleepiness despite the small numbers and that the comparator group, TAU were receiving secondary care interventions. There was also no increase in numbers using rescue medication or indication of important harms in terms of side-effects in either group. The consistency in the direction of all the results suggests they may be real. It is important if our intervention reduces daytime sleepiness, in contrast to sedative medication, which sometimes increases it. Similarly, quality of life is an important outcome for someone with dementia, as an intervention may improve one domain while reducing overall quality of life. Correspondingly, the carers in the intervention group indicated reductions in stress and burden.

The PPI judged the important outcomes were that the person with dementia was less restless at night, more awake during the day, disturbed the carer less and seemed happier (which the SDI measures). In these circumstances, they were unsure that actigraphy added information, or was accurate. Although some carers were disappointed with actigraphy feedback, the carers and therapists found the actigraphy information from baseline helped to delineate the rest-activity pattern and help make a plan. We would continue to incorporate this in the intervention manual in a full trial.

Future trials and interventions require a degree of flexibility about who receives the intervention. We had expected we would deliver an intervention mainly to family carers alone, but the intervention was delivered to family carers, paid carers, and sometimes to both. Frequently, the person with dementia was also included, and in one case half of the sessions were delivered to a person with dementia alone. Delivering the intervention with the person with dementia present, although not always problematic, presented challenges for the therapists; for example, when the person with dementia denied having any difficulties with sleep. One person with dementia had some sessions alone, but could not retain the information and make use of the sessions. In any future trial, we suggest that people with dementia could jointly participating in the intervention sessions, with additional training for the therapists on delivering sessions with people with dementia present and managing any resulting conflict and interpersonal challenges. However, we recommend, only delivering the intervention to people with dementia with a carer also participating.

### Strengths and limitations

We had envisaged that someone with sleep disturbance and dementia would have a paid or family member with them at night to ensure safety. This was not always the case. When people lived alone, the carers (whether family or paid) were unable to implement strategies, such as a scheduled bedtime or wind down routine. It was also difficult to gain reliable information about the sleep patterns of people with dementia living alone. We would therefore suggest excluding people without a night-time carer in a full trial.

When paid carers attended the intervention they were also able to implement strategies. Working with them may be important to allow people to remain living at home – care agencies require full-day rates and two carers, if the carer is disturbed frequently during the night. Since it appeared feasible and is potentially useful, we would include paid carers if people with dementia and their families so wished in a full trial.

The trial recruited people from London only, limiting external validity. Although male care recipients were more likely to refuse, we succeeded in recruiting carers from either gender, a range of age groups, types and severity of dementia, relationships to the care recipient, marital status, and educational backgrounds. In particular, we recruited about 35% of people of minority ethnic status despite most studies finding they are under-represented. A high proportion of carers remained in the study. Although the outcome assessors were masked to outcome, the participants were not. It is possible that some carers might have felt that they had to report a positive result to please the researcher interviewing them, who was not the therapist, but in most cases had met them for screening and baseline assessment. However, the instruments are validated, and in other studies carers have frequently reported no beneficial effect on these measures (Livingston *et al.*, 2017). If the person with dementia lived alone, the carer would have been likely to report their relative's memory of their sleep and this assessment may be less reliable.

This study finds that DREAMS-START is a feasible and acceptable intervention. High fidelity to the intervention suggests it can be reliably delivered by trained, supervised psychology graduates. The evidence from the validated questionnaires suggests there is potential for efficacy in improving sleep disturbance and quality of life of the person with dementia, and for reducing family carers’ stress. We are able to specify primary and secondary outcomes for an efficacy trial and calculate power. This augurs well for a full trial.

## Supplementary material

For supplementary material accompanying this paper visit http://dx.doi.org/10.1017/S1041610218000753. click here to view supplementary material
