# Supplementary material for: DREAMS-START (Dementia RElAted Manual for Sleep; STrAtegies for RelaTives) for people with dementia and sleep disturbances: a single-blind feasibility and acceptability randomized controlled trial
Source: Int Psychogeriatr. 2018 Sep 17;31(2):251–65. doi: 10.1017/S1041610218000753 (PMC6474714; doi:10.1017/S1041610218000753)
Supplement: Supplementary file 1 [file S1041610218000753sup001.docx]

Appendix A

**DREAMS START (Dementia Related Manual for Sleep; Strategies for Relatives)** feasibility and pilot study

HTA project 14/220/06

Sponsors UCL 16/0111
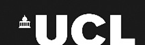


IRAS project 199820

**Background and Rationale**

There are expected to be 850,000 UK residents in 2015 living with dementia, two-thirds of whom live in the community, with numbers increasing rapidly as the population ages^1^. Dementia care costs £26.3 billion (£32,250 per person); £14.6 billion of this comprises health and social care costs.

Sleep disturbances are common in older people. People with dementia may have the same problems as other older people e.g. pain and multiple health conditions, which impair sleep. In addition, the dementia may itself lead to impaired melatonin production, so that people have a decreased regularity of sleep, impaired sleep initiation and continuity, and difficulty maintaining wakefulness during daylight hours. Sleep disturbances in dementia are thus common and varied, including reduced night-time sleeping, night wandering, sleep fragmentation and excessive daytime sleepiness. The cholinesterase inhibitors and glutamate receptor antagonists given to improve cognition, mainly for Alzheimer's disease and Dementia with Lewy Bodies, may also increase wakefulness. It has, however, also been suggested that they help sleep mainly through improved cognition. Sleep subserves all aspects of mental and physical life and is a major determinant of day-to-day function and quality of life^2^. Sleep disturbances occur in 25-40% of those with neurodegenerative dementias^3;4^ but there is no psychological or drug treatment with adequate effectiveness evidence. Sleep disturbances may be caused by and worsen Alzheimer’s disease ^5^ and other dementias, and reduce quality of life for those with dementia. Sleep disorders in this population are distressing both for the person with dementia and their families, with families being particularly distressed by their own sleep being disturbed (moderately to severely distressing to 70% of carers). Sleep disturbance predicts family carer depressive symptoms, increases care burden and leads to care home admission, significantly elevating care costs^6^. Importantly, night-time care costs can be unaffordable for people who wish to continue caring at home. Overall, sleep disturbance affects 210-380,000 people with dementia, their family carers and wider society through increased care costs. There are currently no known effective treatments and health teams respond to need by using a mixture of sleep hygiene measures and psychotropic medication, extrapolated from other conditions which may sometimes cause harm and confer limited benefit.

Evidence explaining why this research is needed now:

The causes of sleep disturbance in individuals with dementia are multifaceted, including physiological dementia-related changes, pain, environmental and behavioural factors, and medication side-effects. A Cochrane review showed no definitive evidence about the benefits of pharmacological treatment of sleep in older people with dementia; and sedative medication may cause harm^7^. Similarly, a recent Cochrane review found that bright light therapy used in this group, without measuring patients’ individual disturbed circadian rhythm, was ineffective^8^.These results are consistent with a forthcoming Cochrane review and a paper from the Liverpool Review and Implementation Group (personal communication). The reviews included studies which examined the effects on sleep of melatonin; the cholinesterase inhibitors, donepezil and galantamine and the antidepressants trazadone and mirtazapine. They find no good evidence for the efficacy of melatonin (given without considering individual’s varying sleep phases) and find that overall there is currently no conclusive evidence about the efficacy of any other drugs.

In addition, people with dementia often are frail with multiple morbidities and non-pharmacological treatment should therefore be first line for sleep management in dementia. However, most evidence about this form of management comes from small scale, often low methodological rigour studies, leading to insufficient, conflicting evidence^9^. The need for better research into non-pharmacological treatments for sleep disorder is mentioned specifically in the National Dementia Strategy (DH, 2009), the outputs of the 2010 Ministerial Dementia Research Summit, and 2011 NIHR Dementia Research Workshop summarised in the Ministerial Advisory Group for Dementia Research (MAGDR) final report “Priority Topics in Dementia Research” published by the MRC in February 2011(DH, 2011). As with many other problems, studies consistently indicate that patients and their doctors would be prefer non-drug approaches for sleep problems.

Our vision is to build on the contradictory and incomplete evidence, bringing together expertise in clinical interventions in dementia and sleep, statistics, and a family carer to develop and test a manual to improve sleep and therefore quality of life for those with dementia and their families. Through our previous work with START, a coping strategy based manual for family carers of people with dementia, delivered via supervised graduate psychologists (including increasing pleasant activities for people with dementia and their family carers), we have successfully demonstrated short-term and long-term clinical and cost-effectiveness^10;11^. This is an ideal platform on which to build our new manual DREAMS (Dementia Related Manual for Sleep).

DREAMS will be a multicomponent intervention, comprising of a cognitive-behavioural component, including coping skills for families and activities for people living with dementia, and light therapy. Dementias are commonly characterised by circadian rhythm disruption because of progressive Suprachiasmatic Nucleus neuronal loss^12^. Thus, strengthening circadian rhythmicity through phototherapy is theoretically appealing. However, light therapy given in a non-individualised way on the wrong side of the phase response curve, for example, may exacerbate disruption. Thus, intervention must be individually tailored using natural dusk and daylight (wherever feasible) as well as other light sources, to manage endogenous melatonin production. We are encouraged that Brown (2013)^9^ found evidence that light therapy, and activity, may be effective. We will use this together with cognitive behavioural sleep management components which a Cochrane review found effective in non-dementing older adults or with family carers of people with dementia. ^13;14^ We will emphasise, as in our previous work, a collaborative, non-prescriptive model, in which we encourage family carers to try techniques and note what works for them in their manual.

The manual will be delivered individually, because sleep disturbances are complex with causes and treatment differing between individuals; and as it can be difficult for family carers to attend a group at a particular time. We intend to train psychology graduates who have a similar skill set to IAPT practitioners and to psychology assistants in memory services and mental health services. This is so that this intervention can be deliverable by IAPT-based psychological wellbeing practitioners and secondary care psychology practitioners. We are currently disseminating START via a “Train the Trainers programme (funded by the Alzheimer’s society). This has included training clinical psychologists and Admiral Nurses embedded within IAPT, memory services and other secondary care older adult services. They are now rolling out the intervention in a range of settings according to available resources and local need, reflecting the differing types and range of service provision for people with dementia and their families.

This research has potential to improve sleep and quality of life of people with dementia and their family carers, in a feasible and scalable intervention without the side-effects of medication. If found to be effective, our intervention should also be cost effective as it is cheap and may also delay care home admission.

**Aims and Objectives:**

Our research question is how feasible is a pragmatic randomised study to investigate the clinical and cost-effectiveness of a manualised intervention (DREAMS START) to manage NHS patients with dementia and significant sleep disturbance living in their own homes.

*Aims*

To develop a manualised behavioural intervention for sleep disorder in dementia and examine feasibility of a full scale trial.

*Objectives*

1. To obtain estimates of acceptability and feasibility that will inform continuation to the main trial. Specifically to estimate (with 95% confidence intervals) the proportion of participants offered the intervention that adhere to it and the proportion of eligible participants who agree to participate in the trial.

2. To obtain estimates required for the main trial’s sample size calculation in relation to potential primary outcomes [standard deviations; correlation between baseline and follow-up measurements and drop-out rate].

3. To use qualitative interviews to assess acceptability of the intervention and to detail any required refinements.

**Design and theoretical/conceptual framework:**

This study encompasses the design of a complex intervention followed by a pragmatic randomised controlled trial for feasibility.

1. Manual development by team (experts in manual development in sleep and in dementia and PPI) using MRC framework for complex interventions, to incorporate existing evidence and qualitative interviews (family carers and people with dementia) about content, clarity, practicality and acceptability.
2. Randomised controlled trial of DREAMS START manual compared to treatment as usual (TAU) for feasibility and acceptability.
3. Qualitative assessment of the experience of the health technology.

**Methods**

**Health technologies being assessed**

*Developing the intervention*

This will be a collaborative development by the team who developed the START manualised intervention (GL, CC, PR) and (CE,SK) experts in manualised cognitive behavioural interventions in sleep disorder and our PPI team who will assist with therapy development and manual refinement.

We will interview family carers and people with dementia, through the Alzheimer’s society, about barriers and facilitators to using our draft manual and how their problems are addressed. This will generate a conceptual framework for understanding how to implement our evidence based intervention. We will develop interview guides based on the research literature, PPI consultation and expert opinion. Questions will be open-ended and revised iteratively to further explore issues raised. Broad topic areas will include the following: understandings of sleep disorder, communication with and about people’s sleep, acceptable activities, support from family, use of actigraph and light therapy acceptability, and impact of sleep deprivation on patient and carer. We will discuss proposed strategies and ask how they would work. Our sampling will be purposive to ensure we cover people with different severities of dementia, differing diagnoses and spouse and non-spouse carers. We will continue conducting interviews until there is theoretical saturation of themes and new themes cease to emerge. Results will feed into the manual and implementation plan with barriers being addressed and facilitators used. The initial manual generated will be shown to family carers and PPI.

*Intervention content*

The health technology being devised and assessed will be the new DREAMS-START manual for family carers, and people with dementia who are able to participate. It will begin with a psychoeducational component for all family carers and those people with dementia who can understand. It will cover sleep and circadian processes and how sleep and brain function change with ageing and dementia.

We expect participants to be seen in their own homes for the intervention. We envisage that the sessions will use practical techniques adapted from the broader evidence-based literature on cognitive-behavioural therapy for poor sleep,^15^ working with family carers of people with dementia and those with dementia who can understand it and plan activities and circadian interventions for sleep-wake disturbance as detailed below. The last session will involve family carers agreeing a future maintenance plan using techniques that were acceptable and successful for them. The family carers will be left with a personalised manual.

Our proposed management strategies will be designed to optimise both the individual’s sleep at night and his/her wakefulness during the day. The timing of the person’s sleep-wake schedule will be established using one week of actigraphy (a non-intrusive, re-usable wristwatch which monitors movement and estimates circadian phase). Information from this will inform shared understanding of the person’s night-time settling and waking problems, and their daytime sleepiness/ fatigue, and help generate an optimal ‘sleep window’. Earlier studies have found actigraphs can be used successfully in this population^16^. To optimise night-time sleep pressure and the circadian regulation of sleep we will work with the carer to deploy practical zeitgebers (cues that influence the person's biological rhythms e.g. regular timing of bed and rising, morning wake-up light, standardised meal times) and to establish adaptive stimulus control (e.g. pre-bed settling routine, management of wakeful episodes). We will also work with the carer on strategies to promote de-arousal and contented and safe wakefulness (e.g. relaxation, bedroom comfort), and help carers to develop coping strategies to manage and address concerns about their own sleep health. During the daytime we will use behavioural activation strategies (e.g. exposure to ambient daylight, outdoor/physical activities, regular timing of meals and indoor routines) to maintain alertness and engagement and to strengthen both central and peripheral clock timing. We will give specific tailored advice about management of daytime naps. Some patients may have pronounced circadian delay or advance, or loss of rhythmicity, which may be less responsive to ambient light and other techniques. For these patients, shifting circadian phase or stabilising a new behavioural rhythm may require using a dawn simulation alarm to anchor the clock in the morning. For those with highly fragmented sleep-wake periods and excessive daytime sleepiness, we will employ alertness promoting light-therapy, at least initially, to help consolidate sleep and wake periods and re-establish rhythmicity. When either of these are the case, we will devise a programme in collaboration with carer and patient around the implementation of individually-tailored phototherapy (using standardised light-box or dawn simulation alarm clocks which increase light gradually). Progress can be measured both by carer report, and objectively through a further one week of actigraphy post-intervention.

Delivery The manual will be delivered individually, both because sleep disturbances are complex with causes and treatment differing between individuals; and as it can be difficult for family carers to attend a group which is only available at one particular time. We will emphasise, as in our previous work, a collaborative, non-prescriptive model, in which we encourage family carers to try techniques and note what works for them in their manual. We intend, as in our previous work, to deliver the intervention as a partnership of psychology graduates and family carers^10^. In practice, most people with dementia in the study will have carers with them at nights, as they will either be referred to services by their family, as causing disturbance to carers sleeping in the same house, or have night care as it is unsafe to leave them alone, but this will not be an inclusion or exclusion criterion. The research assistants will be a supervised psychology graduate, so that the manual can be rolled out using principles of training, supervision and stepped care. Psychology graduates will have a degree in psychology but without clinical psychology training and be recruited on evidence of listening skills, empathy and clinical experience. This level of expertise will ensure breadth of skills (to impart information, knowledge of dementia, mental health & knowing when more support is needed). The psychology graduate research assistants will all complete a specific but short training course, which we will document, to ensure a successful intervention is scalable and affordable, on dementia and sleep-wake regulation/sleep science. In addition, they will complete, UCL training in safety and diversity and training in Good Clinical Practice and informed consent. A short training programme will be delivered by our co-applicants. There will be a strong practical focus on how to deliver the intervention, develop empathic listening skills, make effective use of supervision and when to ask for help. They will also have teaching sessions dedicated to cultural sensitivity. Training will emphasise the need to operate from an inclusive values base and to respect diversity and the existing knowledge and experience of family carers and people with dementia. Knowledge will be acquired through a combination of seminars, discussion groups, reflective learning and guided reading. Skills-based competencies will be learnt through clinical simulation in small groups. In devising our training programme, we will draw on the curriculum for psychological therapists devised by the Department of Health for their improving access to psychological therapies programme (IAPT). All therapists will be trained to adhere to the manual. To ensure treatment integrity, they will be required to demonstrate by role-play, competence in delivering the intervention before recruitment commences. Therapists will, with the carer/patients’ permission, record a random intervention session. They will be supervised by a clinical psychologist (PR) and use recording in supervision. A researcher not involved in the therapy, who will independently rate fidelity to the manual using a standard checklist.

*Treatment as usual*

*Purpose of treatment as usual arm*

We are comparing the new treatment with treatment as usual (TAU) by itself and participants randomised to the new treatment will also receive usual care. The purpose of a treatment as usual arm is to judge acceptability of being randomised, so that we know the feasibility of moving to a full scale trial. In the START trial many of the prospective participants did not agree to take part in the study 181/472 (38%). We did not ask for a reason to refuse to enter the study but many said it was because of randomisation. Of those who did agree to the study, 4/87 (5%) in the TAU group withdrew because they wanted to be in the active treatment group. We are unclear how these figures will translate to this study and wish to test this, although we judge that many people will wish to be in the active plus TAU arm rather than the TAU arm.

*Content of treatment as usual arm*

We will use the Client services Receipt Inventory^17^ to delineate treatment as usual as we have in other studies. We expect it to vary between trusts and also according to individual patient needs but will incorporate the NICE pathways guidelines for dementia. Services are based around the person with dementia. Treatment is medical, psychological and social. Thus, it consists of assessment, diagnosis, risk assessment and information. These include referral to dementia navigators, medication for Alzheimer's disease or dementia with Lewy bodies, cognitive stimulation therapy, START (in some trusts), practical support (social services provided); risk plans, for example telecare, driving information to the Driver and Vehicle Registry Agency (DVLA), medical identification (ID) bracelets, advice regarding power of attorney and capacity assessment; and social services referral for personal care, day centre and financial advice, treatment of neuropsychiatric symptoms and carer support.

**Target population:**

Adults of any age with a clinical diagnosis of dementia and sleep disturbance, living in their own homes

**Inclusion/Exclusion Criteria:**

*Inclusion (all to be satisfied)*

1. Adults with dementia (all types and severities). As this is a feasibility study and there is no theoretical reason to think that this individualised intervention cannot be used in all severities and types of dementia we will not exclude any type or severity of dementia *a priori* but will record these details and use the qualitative assessment to inform us as to whether this should change for the definitive study
2. Sleep Disorders Inventory score ≥4 (a reliable and valid measure of sleep in dementia) ^18^. The Sleep Disorders Inventory is a standalone tool for sleep disorder in people with dementia. It was developed for use as outcome in original melatonin trials and has been used in pharmacological and non-pharmacological studies and has been validated against actigraphy and clinical variables. Those who score ≥4 are judged to have clinically significant sleep disorder.
3. Sleep that patient and their family judge is a problem-this is a pragmatic study and if the symptom is not of concern to the patient or family they will not be included. This is normal clinical practice.
4. Patient gives consent if has capacity or consultee gives consent and patient not unwilling if they are not able to give capacitous consent.
5. Family carer able and willing to give informed consent.
6. Family carer gives emotional or practical support at least weekly to the person with dementia.

*Exclusion criteria (any)*

- Patient living in a care home
- Patient has other sleep disorder diagnosis eg sleep apnoea.
- Family carer not able to give informed consent

**Setting/context**

Patients will be those with a clinical diagnosis of dementia who will be recruited from memory clinics or from any other setting from which they are referred. Assessments and interventions will take place in people’s homes.

**Outcomes**

We envisage the main study’s primary clinical outcome will be measured through actigraphy. This is a non-intrusive, re-usable wristwatch which monitors movement and estimates circadian phase and has been used successfully in trials of people with dementia and sleep disturbance before^16^. The outcome will be indexed through sleep efficiency. This captures both initiation and maintenance of sleep, reflecting proportion of time in bed spent asleep and has been found to be reliably impaired in actigraphy studies of people with dementia and sleep problems. However we will also examine circadian amplitude variables (using nonparametric circadian rhythm analysis) to assess relative amplitude and intradaily stability, since our intervention is also aimed at stabilising sleep-wake patterning and reducing daytime sleep as well as improving night-time sleep consolidation. Our pilot trial will help us decide the future (main trial) outcomes definitively. We will consider outcomes overall in line with recommendations about how to make decisions as to which data can be used to inform the progress to a pragmatic trial and be incorporated within in it^19^.

*Primary outcomes*

Feasibility of recruitment-agreement to study/randomisation.

Treatment Adherence (attending predetermined session numbers-intervention group only)

*Secondary outcomes*

- Referral rates
- Follow up rates
- All psychotropic medication prescription (to define rescue medication’s role),
- Reported side effects: co-morbid physical illnesses and patient falls.
- Choice of outcomes for main trial. We have several measures of sleep (objective and subjective). We will consider feasibility and acceptability by:
  - Completion rates of instruments
  - Acceptability of tools from the qualitative interviews below
  - Estimates of statistical power and sample requirements based on detecting significant differences in outcomes.
- Qualitative assessment of intervention in intervention group only: Post-unblinding, RAs will interview up to 20 carers and patients about the intervention (involvement, practicality, acceptability) and about their opinion of the assessment until theoretical saturation is achieved.

**Data collection**

*Patient / Family Carer Measures (Baseline and three month follow up)*

We will be keeping careful records about eligible referrals, consent, and adherence to intervention as these are the primary measures of feasibility and acceptability.

We will collect all interview data from the carer to reduce patient burden and to ensure that data is comparable if the degree of impairment of the patient prevents them completing the questionnaires (interview time: expected to be about 50 minutes). We will inform feasibility and test procedures by collecting validated clinical and cost effectiveness measures planned for a full trial. We will also, as the HTA requests, ask study participants to consent to long-term follow-up beyond the study using routinely collected data from GP, electronic mental health service notes and DCRIS databases.

Patient: Baseline only

1. Socio-demographic details for the included participants with dementia: age, sex, current marital status, ethnicity, level of education, last occupation (baseline only).
2. Medication- psychotropic medication at baseline and at three months (for three months) to delineate the role of rescue medication (incorporated in CSRI below).
3. Dementia- type diagnosed (from referral)
4. Severity of dementia (Clinical Dementia Rating; CDR ^20^). The CDR has six domains: Memory, Orientation, Judgment and Problem solving, Community Affairs, Home and Hobbies, and Personal Care. It is widely used to assess dementia severity through informants.

Patient: Baseline and three months

1. Sleep Disorders Inventory^18^ is validated for measuring sleep disorder in people with dementia and describes the frequency and severity of sleep-disturbed behaviours
2. Actigraphy (baseline and 3 months)
3. Neuropsychiatric symptoms (Neuropsychiatric Inventory)^21^, a validated instrument with 12 domains, which will be included as a secondary outcome in the main trial . This will enable assessment and consideration of whether other neuropsychiatric symptoms, for example, depression and anxiety, or total neuropsychiatric symptoms, may be changed by the intervention.
4. Epworth sleepiness scale (daytime sleepiness)^22^ to assess changes in excessive daytime somnolence. This is an eight item measure assessing tendency to sleep/doze in specific daily situations (possible score range 0-24; a score of >10 indicating excessive sleepiness).
5. DEMQOL proxy ^23^ is a 31 item interviewer-administered questionnaire answered by a carer. It is responsive, valid and reliable measures of quality of life in people with dementia. They have psychometric properties at least as good as other dementia-specific quality of life instruments^24^ and have been used to generate utility values^25^ .
6. Patient services use (CSRI)^17^ is widely used for dementia trials and will delineate treatment as usual (TAU) as well as treatment for those in the active arm of the study and together with the DEMQOL proxy can be used for cost effectiveness^26^.
7. Side effects measure for fall and comorbidities at baseline and follow up. Using a Safety, and Tolerability Assessment to record the occurrence of falls, dizziness, headaches and gastrointestinal symptoms (appetite or bowel symptoms) and any other side effects and whether these were mild, moderate or severe

Carer: baseline and 3 months

1. Socio-demographic details for the carer: age, sex, relationship to patient, co-residency or if not how often sees patient, ethnicity, last or current occupation (baseline only).
2. We will administer two questionnaires for sleep quality to consider which of these is better in this population
   - 1. Pittsburgh Sleep Quality Index: (PSQI)^27^ a validated, reliable instrument to measure the effect on carer sleep (since it is commonly disrupted by sleep-wake patterning of the person with dementia
     2. Sleep Condition Indicator (SCI)^28^ a new eight item scale developed in the UK with date on tens of thousands of people of all ages, and characterises sleep both dimensionally (like PSQI) but also against insomnia disorder criteria (which PSQI does not).
3. The hospital anxiety and depression scale (HADS) ^29^ is a validated , reliable measure to measure mood in carers throughout the age groups
4. Zarit^30^ the most commonly used and well validated measure of burden for carers of people with dementia to indicate whether burden may be changed by the intervention
5. Carer HSQ^31;32^ a 12 item health-related quality of life scale validated throughout the age group and together with CSRI be used for cost effectiveness.

*Power calculation*

With 40 intervention participants (larger to allow a more precise estimate of proportion adhering to intervention) and 20 controls we will achieve the following 95% confidence intervals (CI) for our expected adherence and participation estimates:

1 Proportion of participants adhering to intervention- expected value 75%, 95% CI: 59-87%

2 Proportion of appropriate referrals consenting to the trial-expected value 50%, 95% CI:41-59%

We judge that these confidence intervals provide acceptable ranges to inform continuation to the main trial. Overall we expect that our “stop-go” measures would be related to the proportion adhering-

1. >=70% - go to main trial
2. 60-69 –consider a modified trial design to increase adherence
3. <60 do not progress to main trial using this model.

This sample size will also be sufficient to estimate the standard deviation required for the sample size calculation in the main trial^33;34^.

We estimate that recruitment referral rate will be approximately six potential participants per week; two participants will be suitable and will agree to participate (one per research assistant); and follow-up will be approximately 80%.

**Assessment**

*Procedures*

Referrals and recruitment procedure

Prospective eligible participants will be initially approached by a clinician they know and given (or sent) an information sheet or will volunteer through Join Dementia Research. Some prospective participants may have agreed to be contacted for research (as trusts may have a register) but we will still approach a clinician initially to ensure there have been no changes and ask, if possible, if they can make contact. Our experience is that this group of patients may not remember consenting and be very cautious of calls from strangers. Those interested in participating will be referred to the research team. The referral will give the name, sex and relationship of the patient and carer (this will allow testing for external validity by comparing the sample recruited with those who were referred but refused recruitment). The team that is to carry out blinded assessments will telephone them 24 hours or more after they receive the information sheet. They will answer any questions and then arrange to meet those who thought they wished to take part, to obtain their informed consent and complete the baseline assessment before randomisation.

Randomisation

We will use a randomisation list produced by the statistician. It will be concealed using sequentially numbered envelopes to be opened by a specified person in the team. As our psychologist will know who is having therapy (as supervisor) she will hold these envelopes.

Blinding

The graduate delivering the intervention in one group will assess the outcome of those in the opposite group blind to randomisation status. Outcome assessors will be blinded to randomisation status, but it will not be possible to blind study participants. The researchers will work separately, each assessing outcomes for approximately half the participants and providing therapy to those allocated to treatment in the half of participants they were not assessing. These teams will have supervision separately so as to remain blinded. Assessors will ask participants at the beginning of each interview not to disclose their allocation group. All assessments will take place in the home of the person with dementia or carer. We will require written consent according to the Mental Capacity Act (see details below)

**Data analysis:**

Randomised controlled trial

Our co-applicant statistician will lead and supervise the analysis. The statistical analyses will be described in a predefined statistical analysis plan, and planned and conducted according to ICH E9 and following the standard operating procedures of the PRIMENT clinical trials unit. A summary of the main analyses are given here.

Baseline data will be summarised by treatment group using means (with standard deviations), medians (with interquartile ranges), counts and proportions, as appropriate, to gauge the balance in characteristics between the randomised groups. A consort diagram will describe the flow of patients through the trial.

1. The proportion of participants in the intervention group that adhere to the treatment (attend a predetermined number of sessions) and the proportion of appropriate referrals consenting to the trial will be calculated with 95% confidence intervals.
2. Actigraph measurements of sleep efficiency (%), total sleep time (minutes) and wakefulness during the sleep period (minutes), the Epworth sleep inventory scores and the Sleep Disorders Inventory Scores will be summarised to provide distributional information and to obtain estimates of standard deviation and correlation needed for the sample size calculation of the main trial
3. Referral rates, drop out and loss to follow up rates will be calculated with 95% confidence intervals
4. In addition, prescription of psychotropic medication (to define rescue medication’s role) and co-morbid physical illnesses and patient falls will be summarised by randomised group.
5. Other patient and carer outcome data will also be summarised by randomised group using appropriate estimates with 95% confidence intervals.

Qualitative study

Interviews will be audio-recorded and transcribed verbatim. The transcribed interviews will be entered into a software package for qualitative data analysis (NVivo). Data from the interviews will be separated into meaningful fragments and labelled with descriptive codes. We will use a thematic and framework approach^35^, systematically coding and annotating transcripts and displaying in matrices and diagrams until we have a comprehensive picture of all the phenomena in question. Thus the analysis will use standard, recommended methods to ensure rigor^36^. Each transcript will be read by two researchers independently, and then ideas regarding coding frames discussed within the team. The constant comparison method will be used to identify similarities and differences in the data. Disagreements between the raters will be resolved by discussion.

**Dissemination and projected outputs:**

This proposal is for the development and feasibility testing of an intervention and it will not yet be at the stage for there to be full dissemination as it will require a full scale randomised controlled trial for effectiveness and cost effectiveness based on its findings. We therefore plan limited dissemination to the academic community, publishing in peer-reviewed, high-impact academic journals, disseminating our findings at national and international academic conferences and working with the Alzheimer's Society for media coverage.

We envisage that if this work progresses to a full trial it will be embedded in an extensive network of academic researchers, policy makers, stakeholders and PPI, acting as a ‘community of interest’ to advise on and ensure acceptability for the dissemination of the research, and also to ensure that this reaches both the national and international communities. This group meeting will be arranged every six months and the trial manager will co-ordinate a report for the group from all workstream leads. Members of this community will include; Alzheimer’s society [AS], Dementia UK, UCLPartners, Research networks, and International Longevity Centre (ILC). The group will provide external advice and scrutiny throughout the process, ensuring the project is situated in current policy and practice and provide a roadmap for future action and development and be written accessibly for both politicians and a wider lay audience and in turn disseminated to a wider audience. The ILC are experienced national government lobbyists, whom we currently work with and will lead dissemination of a full scale project and launch the major outputs to press, MPs, the House of Lords, voluntary sector and prominent scientists.

While this is only a pilot, and a full trial would be needed before implementation, we do have a plan for this. We have recruited Judy Leibowitz, head of Camden and Islington IAPT, as a collaborator and member of the steering committee to ensure our manual is suitable for IAPT. We would also apply for an Alzheimer’s Society dissemination grant to kick-start a “Train the Trainers” programme (as we have for START) and an implementation grant. We would “Train the Trainers” so that a clinical psychologist or Admiral nurse could train and supervise people to deliver this either through IAPT or through the psychology assistants in memory clinics and other secondary care older adult services who will roll out the intervention. The leader manual for facilitators will have details about how to deliver and vary the intervention according to people’s needs. This intervention would fit into post diagnostic pathways which are already set up, utilising an existing workforce skilled in delivering psychosocial interventions to people with dementia and their carers

Thus there is an urgent need for an effective management package for people with dementia and sleep difficulty. We anticipate that our research will produce a package which is acceptable and potentially scaleable and will have a very large impact on reducing distress for people with dementia and their family carers, and potential economic effects by delaying care home admission.

**Plan of investigation and timetable:**

Workpackage 1 (WP; 6 months)

01.02.16- 31.07.2016

-1 to 0 month: Place advert for psychology assistants with date of interview

0-1 month: Shortlist, interview and offer post, two RA to start June and one manager at the beginning of May if possible

0-6 months: Sponsorship, ethics and governance approvals, research passports applied for and put in place. The application for research ethics committee approval will be prepared by the CI and reviewed and signed by the sponsors (UCL) prior to submission. Local NHS Trust R&D site approvals and ethics committee approvals will be facilitated by CRN DeNDRoN.

0-6 months: Co-applicants develop manual using MRC framework for complex interventions, to incorporate existing evidence and qualitative interviews (family carers and people with dementia) about content, clarity, practicality and acceptability. We will also devise a training programme.

4-6 months: First RA joins in month 4 to help production of the final manual version; set up database for referrals; for assessments and separate password protected databases for each RA to keep data about intervention patients including adherence, time taken in phone calls and therapy and produce final case record forms.

4-6 months. Other two RAs join in month 6. Begin to train psychology graduates as research assistants (RA) and in the manual.

WP 2 (10 months) 01.08.2016- 31.05.2017

Using our previously tested recruitment techniques, RAs and research networks recruit 60 participants from memory clinics. Those assessing participants will be blinded to randomisation status. We will randomise individually to intervention or TAU asking participants not to tell assessors. Those randomised to intervention will receive usual care plus the intervention. Participants will be followed-up at three months. Post-unblinding, RAs will interview up to 20 carers or people with dementia about the intervention (involvement, practicality, acceptability) until theoretical saturation is reached. We envisage the changes will be relatively small as the process of the intervention development will have ironed out most of the modifications needed in our development phases. We expect to make small modifications in the final interview. As above , we will invite original members of the focus group to comment on the qualitative interview data, consider revisions and review the final manualised intervention (see last question). We think these will be similar to when we devised START when the modifications after the intervention were to spell out instructions to the facilitators. These included:

• With regards to repetitiveness – people get ideas more or less quickly. Leader will adapt and cope with repetition in manual

• Structure of manual – this will fit for most people. But if the order needs to be changed to feel more relevant for them, it can be. This can be flexible, try to tailor for individual. There may be a particularly pressing question that needs to be addressed. Behaviour should come before cognitive.

• Where to see carer – should be identified in pre-session assessment.

• Need to overtly explain the benefits of doing homework in the intro / first session – then reminding throughout sessions. Making the benefits more explicit and phrasing them as beneficial to them

WP 3 (5 months)

Data clean

Analysis (RA finish qualitative analysis and statisticians analyse quantitative data).

Write up of detailed HTA report and draft of paper

**Co-applicant team**

Gill Livingston [GL; chief investigator] and Claudia Cooper [CC] are clinical academics in memory clinics). They with co-applicant Penny Rapaport [PR] have experience in manual based therapies for people with dementia and have devised and tested the successful multi-domain START coping intervention (including activity) for family carers in the NHS and are now disseminating it into UK practice ^10;11^. Julie Barber [ JB]) is a statistician and works in a clinical trials unit PRIMENT and has analysed START. All four of these co-applicants are working together on the MARQUE study This includes devising and testing the manualised MARQUE intervention for reducing agitation in people with dementia in care homes as part of the ESRC/NIHR funded Prime Minister’s dementia challenge

Colin Espie (CE) and Simon Kyle (SK) are specialists in sleep research both in understanding mechanisms by which behavioural interventions modify sleep and circadian rhythms, and in successful translation to clinical practice ^2;15;28;37-39^.

We are partnered by Alzheimer's Society (AS) who are leading PPI. James Pickett, head of research at the AS will be our overall PPI lead and Rossana Horsley who has lived experience as a carer and is a member of the Alzheimer’s Society Volunteer Network will continue her input

**Funding**

This study is funded by National Institute for Health Research HTA Project:14/220/06

**Project management**

The project will be sponsored by UCL and will require ethics and research and development approvals.

GL will have day to day management and control of the project and the project development group will meet at the beginning of the project and will outline the development of the manual and the tasks involved and meet weekly by teleconference or in person to ensure continued progress and milestones. The research assistants will have individual clinical supervision fortnightly by PR and will have the opportunity to consult GL, CC or PR in between as required. The co-applicants will comprise the Trial Management Group (TMG) and will meet three monthly throughout the project. There will be an independent Trial Steering Group (TSC) with PPI and IAPT service contribution. Trial Management and Steering Committees will ensure adherence to the Mental Capacity and Data Protection Acts; ethical guidelines and Information Governance procedures.

Central Monitoring

Each site to email the sponsor twice yearly:

1. Delegation log
2. Adverse Event log
3. Deviation Log
4. Minutes of trial Steering Committee (for equivalent)
5. Annual progress report (lead site only) when sent to Ethics Committee.

Information Governance

UCL processes the personal data of living individuals such as its research subjects in line with the Data Protection Act 1998 (DPA). The UK’s regulator for the DPA is the Information Commissioner’s Office. It is the duty of data controllers such as UCL to comply with the data protection principles with respect to personal data. All electronic data will be stored without name or address. Data will be held on a secure database on a password-protected UCL computer. Access to data will be restricted to the research team. To enable follow-up, it will be necessary to keep contact details for the participants, but this information will be restricted to key members of the team. Audio- recordings of interviews for training and fidelity monitoring (using a digital voice recorder) will be destroyed once the main study is complete. The digital recordings from the qualitative study and process evaluations will be destroyed when the study is completed.

Clinical Trials Regulations

As a psychological intervention, this trial is exempt from registration under the Medicines for Human Use (Clinical Trials) Regulations 2004. We will register the trial with the appropriate body (www.controlledtrials.com) and assigned an ISRCTN number in accordance with good practice.

**Approval by ethics committees**

Ethics committee GL will fill in ethical application at the commencement of the study (while developing the manual) and if the ethics committee insists on seeing the final manual (our experience is they are inconsistent) we will ask for approval for the qualitative interviews and then submit the manual as a major amendment for speed of approval. We will go to an appropriate full Research Ethics committee through IRAS and recruitment will not commence until permission is given. UCL will be the projects sponsors. Research and Development will be led by NoCLoR, the Research and Development Organisation of the lead trust.

The project will use information sheets and these will be given 24 hours or more in advance to those who are asked to give consent. All those participating will give written, informed consent if they can and if not able we will use consultees in accordance with the Mental Capacity Act. All data will be anonymised. Confidentiality will be kept unless there is evidence of harm to vulnerable people. This will be specified in the information sheet. All electronic data will be stored without name or address. Data will be held securely on a password-protected UCL computer. Access to data will be restricted to the research team. To enable follow-up, it will be necessary to keep contact details for the participants, but this information will be restricted to key members of the team. Audio- recordings of interviews for training and fidelity monitoring (using a digital voice recorder) will be destroyed once the main study is complete. The digital recordings from the qualitative study and process evaluations will be destroyed when the study is complete

Capacity of participants to consent

We will adhere to the Mental Capacity Act (2005) when interviewing people with dementia who do not have capacity. The potential participants' agreement to participate will be obtained to their best level of understanding and recruitment will not proceed if they refuse or show significant distress. For those without capacity consent will be requested from the potential participant's personal representative, their main family carer, who would have best knowledge of the individual's attitudes and stated preference to research and consequently best placed to judge whether they would have wished to participate if they had capacity.

Safeguarding

If information disclosed during the study leads us to believe that a patient or carer is at risk, the researcher will discuss this with their supervisor. The information sheet will say "we respect confidentiality but cannot keep it a secret if anyone is being seriously harmed or is at high risk of serious harm". If there is reason to believe that harm is occurring to a vulnerable person or there is a high risk it is likely to occur, we will report this to the appropriate clinical manager with or without the consent of the carer, if this is refused. If the information is reported by a person with dementia we would proceed as above, but in consultation with supervisors who would if necessary see the person with dementia. If the harm is to the family carer, we would consider whether that person had capacity to decide whether they wanted the abuse to be reported.

**Patient and Public Involvement**

We judge that research with meaningful, proportionate involvement is likely to be of higher relevance and ethical quality. This involvement has already helped us define the research more clearly, eg with questions being posed as to whether family paying for a carer at nights would exclude someone (it will not) and to clarify our lay communication. Our PPI input was integral to all stages of the grant preparation, and will continue throughout. Our PPI rationale is to ensure the presence of the family carer and people with dementia’s voice throughout the study from design onward so the research is meaningful and quality optimised. The protocol and methods were devised in partnership with our AS representatives and DEnDRoN. They will help ensure that our intervention is a patient and carer centred programme of care for sleep disturbance that has the potential to benefit both patients and their carers

We are partnered by Alzheimer's Society (AS) who are leading PPI. James Pickett, head of research at the AS will be our overall PPI lead and Rossana Horsley who has lived experience as a carer and is a member of the Alzheimer’s Society Volunteer Network will continue her input

PPI is envisaged throughout the study, particularly during the intervention development. This study also builds on a wealth of user knowledge and PPI from development of the START manual.

We will tell the Alzheimer’s Society network (>260 people with a personal experience of dementia -carers, former carers and some people with dementia) of this study. We expect that between 12-20 will be interested and we will recruit them to one of two focus groups. The groups will explore the issues faced by carers around sleep, solutions, barriers and considerations about developing feasible and acceptable interventions. Participants will receive small gift tokens for their input. They will be asked if they wish to continue involvement, and we anticipate forming a small group to work iteratively with the team developing the intervention.

Later stages of the study involve qualitative feedback from participants trialling the intervention. We will invite the focus group to consider revisions and review the final manualised intervention.

Our PPI representatives will be on our Trial Management Group and on our steering group to provide partnership, discuss with others in the AS, enhance the relevance, practicality and understandability of our materials and be partners in dissemination. A second carer will be invited to join the project management group with Rossana Horsley, a co-applicant, already involved in the design of the study. They will advise on the language and content of information sheets and the intervention. They will help ensure that our intervention is a patient and carer centred programme of care for sleep disturbance that has the potential to benefit both patients and their carers. We will follow FACTOR guidance on supporting carers. Training and support costs have been incorporated into the proposal. The AS will provide training and support for our PPI and we will encourage attendance at the one day PPI course at UCL and research network training. The AS will also help with dissemination of our findings, both in terms of the lay summary and in communication with the AS members and more widely.

References

(1) Prince M, Knapp M, Guerchet M, Mccrone P, Prina M, Comas Herara A et al.

Dementia UK: Update . [2]. 2014. Alzheimer's Society 2014. 29-12-2014.

Ref Type: Online Source

(2) Kyle SD, Espie CA, Morgan K. "...Not Just a Minor Thing, It Is Something Major, Which

Stops You From Functioning Daily": Quality of Life and Daytime Functioning in Insomnia. Behavioral

Sleep Medicine 2010; 8(3):123-140.

(3) Moran M, Lynch CA, Walsh C, Coen R, Coakley D, Lawlor BA. Sleep disturbance in

mild to moderate Alzheimer's disease. Sleep Medicine 2005; 6(4):347-352.

(4) Dauvilliers Y. Insomnia in patients with neurodegenerative conditions. Sleep

Medicine 2007; 8:S27-S34.

(5) Ju YES, Lucey BP, Holtzman DM. Sleep and Alzheimer disease pathology-a

bidirectional relationship. Nature Reviews Neurology 2014; 10(2):115-119.

(6) McCrae CS, Dzierzewski JM, McNamara JP, Vatthauer KE, Roth AJ, Rowe MA.

Changes in Sleep Predict Changes in Affect in Older Caregivers of Individuals with Alzheimer's

Dementia: A Multilevel Model Approach. J Gerontol B Psychol Sci Soc Sci 2014.

(7) McCleery J, Cohen D, Sharpley A. Pharmacotherapies for sleep disturbances in

Alzheimer's disease. Cochrane Databaseof Systematic Reviews 2014;(3).

(8) Forbes D, Blake C, Thiessen E, Peacock S, Hawranik P. Light therapy for improving

cognition, activities of daily living,

sleep, challenging behaviour, and psychiatric disturbances in

dementia. CD003946. DOI: 10.1002/14651858.CD003946.pub4. 2014. Cochrane Database of

Systematic Reviews.

(9) Brown CA, Berry R, Tan MC, Khoshia A, Turlapati L, Swedlove F. A critique of the

evidence base for non-pharmacological sleep interventions for persons with dementia. Dementia

(London) 2013; 12(2):210-237.

(10) Livingston G, Barber J, Rapaport P, Knapp M, Griffin M, King D et al. Clinical

effectiveness of a manual based coping strategy programme (START, STrAtegies for RelaTives) in

promoting the mental health of carers of family members with dementia: pragmatic randomised

controlled trial. BMJ 2013; 347:f6276.

(11) Livingston G, Barber J, Rapaport P, Knapp M, Griffen M, King D et al. Long-term

clinical and cost-effectiveness of psychological intervention for family carers of people with

dementia: a single-blind, randomised, controlled trial. Lancet Psychiatry 2014; 1(7):539-548.

(12) van Someren EJ, Hagebeuk EE, Lijzenga C, Scheltens P, de Rooij SE, Jonker C et al.

Circadian rest-activity rhythm disturbances in Alzheimer's disease. Biol Psychiatry 1996; 40(4):259-

270.

(13) Montgomery P, Dennis J. Cognitive behavioural interventions for sleep problems in

adults aged 60+. Cochrane Database Syst Rev 2002;(2):CD003161.

(14) Sivertsen B, Nordhus IH. Management of insomnia in older adults. Br J Psychiatry

2007; 190:285-286.

(15) Bothelius K, Kyhle K, Espie CA, Broman JE. Manual-guided cognitive-behavioural

therapy for insomnia delivered by ordinary primary care personnel in general medical practice: a

randomized controlled effectiveness trial. J Sleep Res 2013; 22(6):688-696.

(16) McCurry SM, Gibbons LE, Logsdon RG, Vitiello MV, Teri L. Nighttime insomnia

treatment and education for Alzheimer's disease: a randomized, controlled trial. J Am Geriatr Soc

2005; 53(5):793-802.

(17) Tractenberg RE, Singer CM, Cummings JL, Thal LJ. The Sleep Disorders Inventory: an

instrument for studies of sleep disturbance in persons with Alzheimer's disease. J Sleep Res 2003;

12(4):331-337.

(18) Charlesworth G, Burnell K, Hoe J, Orrell M, Russell I. Acceptance checklist for clinical

effectiveness pilot trials: a systematic approach. BMC Med Res Methodol 2013; 13:78.

(19) Morris JC. Clinical dementia rating: a reliable and valid diagnostic and staging

measure for dementia of the Alzheimer type. Int Psychogeriatr 1997; 9 Suppl 1:173-176.

(20) Cummings JL, Mega M, Gray K, Rosenberg-Thompson S, Carusi DA, Gornbein J. The

Neuropsychiatric Inventory: comprehensive assessment of psychopathology in dementia. Neurology

1994; 44(12):2308-2314.

(21) Johns MW. Sleepiness in different situations measured by the Epworth Sleepiness

Scale. Sleep 1994; 17(8):703-710.

(22) Smith SC, Lamping DL, Banerjee S, Harwood RH, Foley B, Smith P et al. Development

of a new measure of health-related quality of life for people with dementia: DEMQOL. Psychol Med

2007; 37(5):737-746.

(23) Smith SC, Lamping DL, Banerjee S, Harwood RH, Foley B, Smith P et al. Development

of a new measure of health-related quality of life for people with dementia: DEMQOL. Psychological

Medicine 2007; 37(5):737-746.

(24) Mulhern B, Smith SC, Rowen D, Brazier JE, Knapp M, Lamping DL et al. Improving the

measurement of QALYs in dementia: developing patient- and carer-reported health state

classification systems using Rasch analysis. Value Health 2012; 15(2):323-333.

(25) Beecham J KM. Costing psychiatric interventions. In: Thornicroft G, editor.

Measuring Mental Health NeedsCosting psychiatric interventions. 2 ed. London: Gaskell; 2001. 200-

224.

(26) Rowen D, Mulhern B, Banerjee S, Hout B, Young TA, Knapp M et al. Estimating

preference-based single index measures for dementia using DEMQOL and DEMQOL-Proxy. Value

Health 2012; 15(2):346-356.

(27) Buysse DJ, Reynolds CF, III, Monk TH, Berman SR, Kupfer DJ. The Pittsburgh Sleep

Quality Index: a new instrument for psychiatric practice and research. Psychiatry Res 1989;

28(2):193-213.

(28) Espie CA, Kyle SD, Hames P, Gardani M, Fleming L, Cape J. The Sleep Condition

Indicator: a clinical screening tool to evaluate insomnia disorder. BMJ Open 2014; 4(3):e004183.

(29) Zigmond AS, Snaith RP. The hospital anxiety and depression scale. Acta Psychiatr

Scand 1983; 67(6):361-370.

(30) Zarit SH, Reever KE, Bach-Peterson J. Relatives of the impaired elderly: correlates of

feelings of burden. Gerontologist 1980; 20(6):649-655.

(31) Barry TL, Kaiser KL, Atwood JR. Reliability, validity, and scoring of the Health Status

Questionnaire-12 version 2.0. J Nurs Meas 2007; 15(1):24-35.

(32) Pettit T, Livingston G, Manela M, Kitchen G, Katona C, Bowling A. Validation and

normative data of health status measures in older people: the Islington study. Int J Geriatr Psychiatry

2001; 16(11):1061-1070.

(33) Sim J, Lewis M. The size of a pilot study for a clinical trial should be calculated in

relation to considerations of precision and efficiency. J Clin Epidemiol 2012; 65(3):301-308.

(34) Julious SA. Sample size of 12 per group rule of thumb for a pilot study.

Pharmaceutical Statistics 2005; 4(4):287-291.

(35) Miles M, Huberman A. Qualitative Data Analysis: an expanded sourcebook. 2nd ed.

California: Thousand Oaks, Sage; 1994.

(36) Livingston G, Leavey G, Manela M, Livingston D, Rait G, Sampson E et al. Making

decisions for people with dementia who lack capacity: qualitative study of family carers in UK. British

Medical Journal 2010; 341.

(37) Espie CA, Macmahon KM, Kelly HL, Broomfield NM, Douglas NJ, Engleman HM et al.

Randomized clinical effectiveness trial of nurse-administered small-group cognitive behavior therapy

for persistent insomnia in general practice. Sleep 2007; 30(5):574-584.

(38) Espie CA, Fleming L, Cassidy J, Samuel L, Taylor LM, White CA et al. Randomized

controlled clinical effectiveness trial of cognitive behavior therapy compared with treatment as usual

for persistent insomnia in patients with cancer. J Clin Oncol 2008; 26(28):4651-4658.

(39) Espie CA, Inglis SJ, Tessier S, Harvey L. The clinical effectiveness of cognitive

behaviour therapy for chronic insomnia: implementation and evaluation of a sleep clinic in general

medical practice. Behav Res Ther 2001; 39(1):45-60.
